# Ischemic Conditioning Protects from Axoglial Alterations of the Optic Pathway Induced by Experimental Diabetes in Rats

**DOI:** 10.1371/journal.pone.0051966

**Published:** 2012-12-20

**Authors:** Diego C. Fernandez, Laura A. Pasquini, Damián Dorfman, Hernán J. Aldana Marcos, Ruth E. Rosenstein

**Affiliations:** 1 Laboratory of Retinal Neurochemistry and Experimental Ophthalmology, Department of Human Biochemistry, School of Medicine, University of Buenos Aires/CEFyBO, CONICET, Buenos Aires, Argentina; 2 Laboratory of Histology, School of Medicine, University of Morón, Buenos Aires, Argentina; 3 Department of Biological Chemistry and Institute of Chemistry and Biological Physicochemistry, IQUIFIB, School of Pharmacy and Biochemistry, University of Buenos Aires, CONICET, Buenos Aires, Argentina; 4 Laboratory of Histology, School of Science, University of Belgrano, Buenos Aires, Argentina; University of Sydney, Australia

## Abstract

Diabetic retinopathy is a leading cause of blindness. Visual function disorders have been demonstrated in diabetics even before the onset of retinopathy. At early stages of experimental diabetes, axoglial alterations occur at the distal portion of the optic nerve. Although ischemic conditioning can protect neurons and synaptic terminals against ischemic damage, there is no information on its ability to protect axons. We analyzed the effect of ischemic conditioning on the early axoglial alterations in the distal portion of the optic nerve induced by experimental diabetes. Diabetes was induced in *Wistar* rats by an intraperitoneal injection of streptozotocin. Retinal ischemia was induced by increasing intraocular pressure to 120 mm Hg for 5 min; this maneuver started 3 days after streptozotocin injection and was weekly repeated in one eye, while the contralateral eye was submitted to a sham procedure. The application of ischemia pulses prevented a deficit in the anterograde transport from the retina to the superior colliculus, as well as an increase in astrocyte reactivity, ultraestructural myelin alterations, and altered morphology of oligodendrocyte lineage in the optic nerve distal portion at early stages of experimental diabetes. Ischemia tolerance prevented a significant decrease of retinal glutamine synthetase activity induced by diabetes. These results suggest that early vision loss in diabetes could be abated by ischemic conditioning which preserved axonal function and structure.

## Introduction

Diabetic retinopathy (DR) is one of the most common and feared complications of diabetes. This disease is a leading cause of blindness in people of working age in industrialized countries and affects the daily lives of millions of people. Vision loss mainly occurs due to chronic hyperglycemia, vascular damage and leakage, edema formation, capillary basement membrane thickening, neovascularization, hemorrhage, and ischemia [1]. Although DR has been considered to be a microcirculatory disease of the retina, it is becoming increasingly clear that neuronal cells of the retina also are affected by diabetes, resulting in dysfunction and even degeneration of some neuronal cells [2]. These neurodegenerative changes include increased apoptosis of retinal ganglion cells (RGCs), glial cell reactivity, microglial activation, and altered glutamate metabolism [3]. Bloodworth et al. [4] proposed that DR is not just a vascular disease, but is a multifactorial disease involving retinal neurons and glia. In agreement, neuronal cell apoptosis [5] and glial dysfunction [6] have been reported in the retinas of diabetic patients. We have recently shown a significant loss of ganglion cell layer (GCL) cells and an increase in the number of apoptotic RGCs 15 weeks after diabetes induction by streptozotocin (STZ) injection [7].

Although retinal microcirculatory abnormalities and neurodegeneration could explain some of the visual deficit induced by diabetes, visual function disorders have been demonstrated in diabetics with very early retinopathy or even before the onset of retinopathy. Psychophysical methods and electrophysiologic measurements indicate abnormalities in visual pathway function [8], such as color vision defects [9]–[12], reduction of pattern electroretinogram amplitude [13]–[14], as well as conduction delays of visual evoked potentials (VEPs) [15]–[18]. It was suggested that impaired VEPs are due to an early involvement of nervous conduction in the optic nerve (ON) [17], supporting that diabetic neurosensory disorders may be due not only to subclinical retinal vascular changes, but also to those in the ON or higher visual pathway. In a STZ-induced diabetic model, we have recently demonstrated alterations at the distal portion of the ON at an early stage of diabetes induction [7]. In that sense, we showed a deficit in the anterograde transport from the retina to the superior colliculus (SC) 6 weeks after STZ injection, a time point at which morphological studies do not reveal RGC loss or substantial alterations in the SC. At this time point, a large increase in astrocyte reactivity occurs in the ON distal (but not intraorbital) portion, which coincides with a significant axon loss. Moreover, profound myelin alterations and altered morphology of oligodendrocyte (OL) lineage are observed at the ON distal (but not proximal) portion [7]. These results suggest that axoglial alterations at the distal portion of the ON could be the first structural change in the diabetic visual pathway. Thus, the development of resources to protect the ON against diabetic damage is a goal of vast clinical importance.

Retinal chronic ischemia is one of the DR hallmarks. At present, there is no effective treatment against retinal ischemic injury. However, it is possible to activate an endogenous protection mechanism that protects from retinal ischemic damage by ischemic pre-conditioning (IPC) [19] or post-conditioning (PostC) [20], which require a brief period of ischemia applied before or after ischemic injury, respectively. Ischemic conditioning does not produce any damage *per se*, and trigger yet incompletely described mechanisms that result in retinal tolerance to the damaging ischemic event. Besides the effect of ischemic conditioning against an acute ischemic episode, brief retinal ischemia pulses significantly prevent retinal functional, morphological, and vascular alterations induced by experimental diabetes [21]. A considerable body of literature strongly support that induction of ischemic tolerance can protect many cellular types (including neurons) against severe ischemic damage. Moreover, a recent study shows that neuroprotective mechanisms can also be operative at synaptic terminals [22]. However, there are no previous reports regarding the ability of ischemic tolerance to protect axons in the central nervous system. Reasoning that the beneficial effects of metabolic stressors such as mild ischemia might protect cell bodies and synaptic terminals, we examined the effect of ischemic tolerance on the early distal axoglial alterations induced by experimental diabetes.

## Results

Table 1 summarizes the average weight and blood glucose levels after the injection of vehicle or STZ. At 6 weeks post-injection, a significant weight loss and an increase in blood glucose levels were observed in STZ-treated rats, as compared with vehicle-injected rats. The weekly application of 5-min ischemia pulses did not change these parameters in diabetic animals.

**Table 1:** Average body weight and blood glucose concentration assessed at different time points.

	Average body weight (g)			Average of blood glucose concentration (mg/dl)		
Time after vehicle or STZ-injection	Control	Diabetes without ischemia pulses	Diabetes with ischemia pulses	Control	Diabetes without ischemia pulses	Diabetes with ischemia pulses
3 days	321.8±13.7	323.3±9.9	318.5±8.9	116.1±14.2	466.1±12.9**	452.7±18.4**
6 weeks	446.1±15.1	319.1±18.4**	314.1±20.6**	105.4±9.4	505.6±10.2**	509.8±17.5**

The injection of STZ induced a significant decrease in body weight and an increase in blood glucose levels. Ischemia pulses in STZ-injected rats did not change these parameters. Data are mean ± SEM (n = 12 animals/group).

** p<0.01 vs. aged-matched control animals, by Tukeýs test.

**Figure 1:**
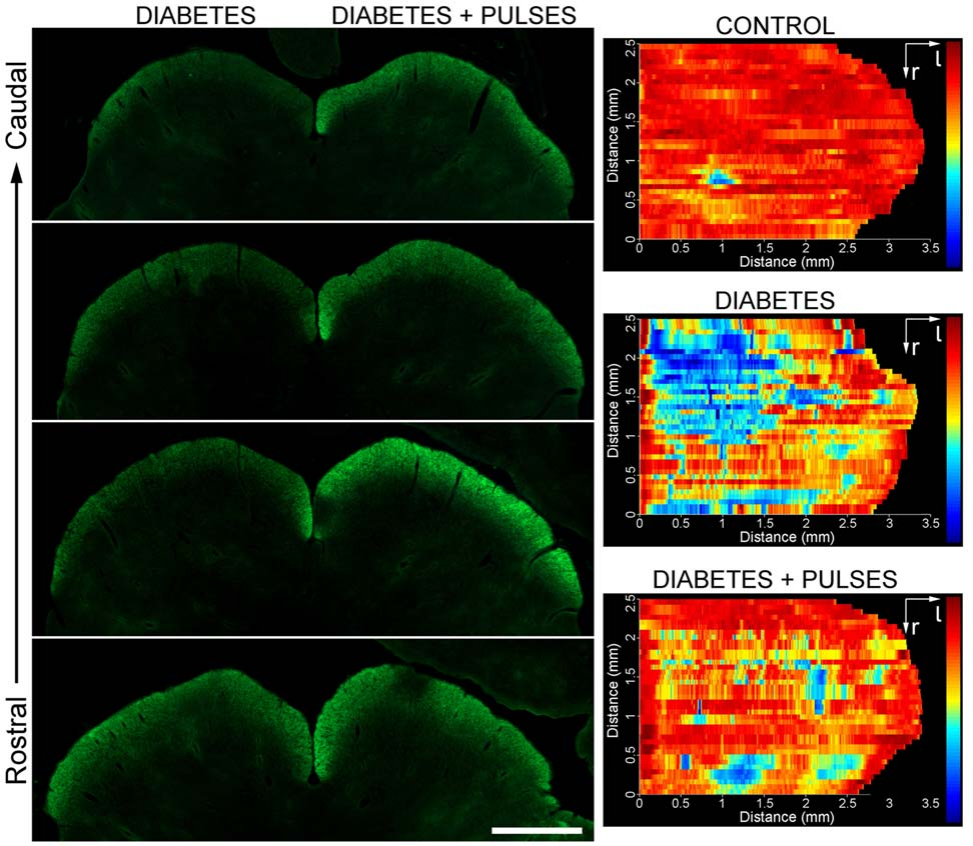
Anterograde transport of CTB from the to the SC. Shown are four coronal sections (from rostral to caudal region) of the SC from a representative control animal (A) or a diabetic animal (B) that received weekly ischemic pulses in one eye and a sham treatment in the contralateral eye. C-E: dorsal view of retinotopic SC map reconstructions from a control (non-diabetic, C) and a diabetic animal submitted to a sham procedure in one eye and ischemia pulses in the contralateral eye are shown. A clear decrease in the CTB-staining pattern in the superficial layers of the SC was observed in the SC that received retinal terminals from a sham-treated eye (D), as compared with the contralateral SC (that received input from the conditioned eye, E). Shown are images representative of five animals per group. Scale bar = 1 mm.

The active anterograde transport of RGC projections to the SC at 6 weeks of diabetes induction was analyzed using CTB. In rodents, virtually all RGCs project to the contralateral SC, particularly to the *stratum zonale* (SZ) and the *stratum griseum superficiale* (SGS), the most superficial layers of the SC. In control animals, intense CTB-stained retinal projections were observed in the SC (Figure 1A). Serial sections were used for a complete reconstruction of the SC in dorsal view. The CTB-intensity was quantified for each section and a thermal scale was finally applied for a clear representation of the CTB-staining pattern. A complete innervations pattern (red: 100% intensity) observed in control animals is shown in Figure 1C. After 6 weeks of diabetes onset, a clear reduction in CTB-staining was observed in the central and temporal region of the SC, extending in rostral to caudal direction (Figure 1B, D). Ischemic conditioning (i.e. weekly retinal ischemia pulses) prevented the deficit in the CTB transport induced by experimental diabetes (Figure 1B, E).

**Figure 2:**
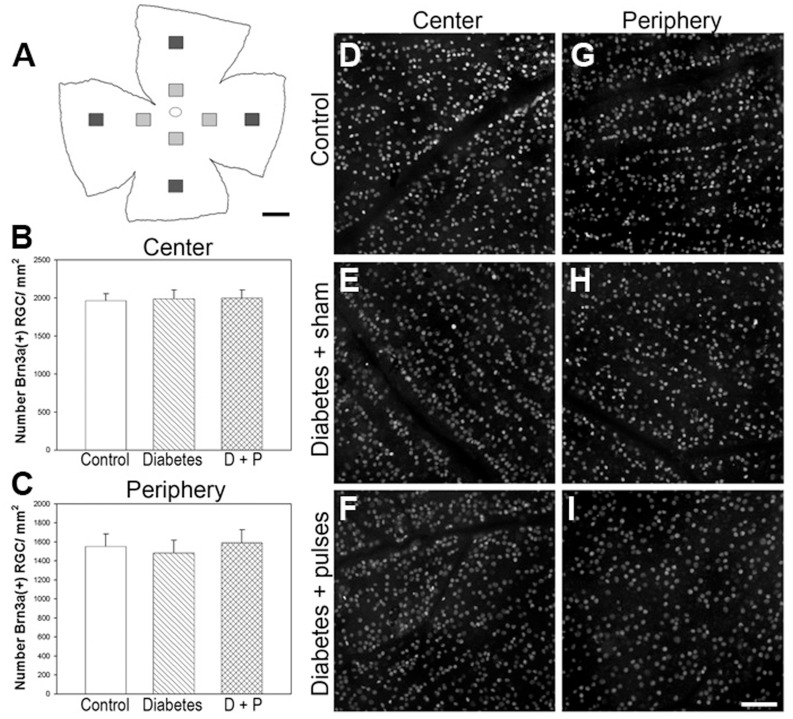
Analysis of Brn3a(+) RGCs in flat-mounted retinas. A: Schematic diagram of a flat-mounted retina showing all the analyzed regions. In central (B) and peripheral (C) retina, the number of Brn3a(+) cells did not differ among groups. Data are mean ± SEM (n = 5 eyes/group, by Tukey’s test). Shown are representative micrographs obtained from flat-mounted retinas form a control (D, G) and a 6-week diabetic eye without (E, H) or with (F, I) ischemia pulses. Scale bar = A: 1 mm; I: 50 µm. D+P: diabetes+pulses.

Figure 2 shows a Brn3a (a specific marker of RGCs) immunostaining study in flat-mounted retinas from non-diabetic eyes and 6-week diabetic eyes submitted to a sham procedure or ischemia pulses. The number of Brn3a(+) cells in the peripheral or central retina did not differ among groups. In addition, total retina, inner plexiform layer (IPL), inner nuclear layer (INL), and outer nuclear layer (ONL) thickness did not differ among groups, as shown in Table 2.

**Table 2:** Morphometric analysis after 6 weeks of diabetes induction.

	Total	IPL	INL	ONL
Control	139.5±18.2	40.2±5.3	19.4±3.1	37.2±4.6
Diabetes+sham	157.8±22.5	44.2±7.2	22.3±6.5	38.6±7.3
Diabetes+pulses	148.2±25.1	42.1±6.4	20.3±1.8	34.7±5.4

Total retinal, inner plexiform layer (IPL), inner nuclear layer (INL) and outer nuclear layer (ONL) thickness (in µm) in control eyes, and eyes from diabetic animals without or with ischemic conditioning. These parameters did not significantly differ among groups. Data are the mean ± SEM (n = 10 eyes per group).

**Figure 3:**
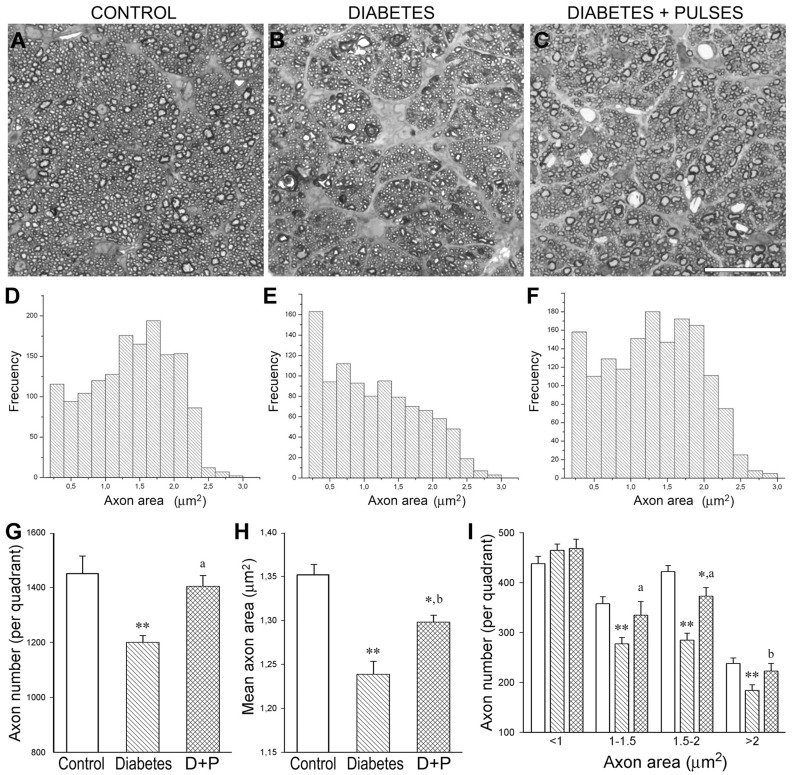
Morphometric analysis of the myelinated distal ON portion. Light micrographs of semithin transverse sections at the distal ON portion from a control rat (A) and a 6-week diabetic rat without (B) or with ischemic conditioning (C) are shown in the upper panel. D-F: Size-frequency histograms showing the axon area distribution at the distal ON portion. In the diabetes+sham group, a significant reduction in the total axon number (G) and in the mean axon area (H), particularly affecting large axons (I), was found. The application of ischemic pulses prevented these alterations. No differences in the ON transversal area were found among groups. Data are mean ± SEM (n = 6 nerves/group); * p<0.05, ** p<0.01 versus age-matched controls, b p<0.05, a p<0.01 versus diabetic ON from sham-treated eyes, by Tukey’s test. Scale bar = 25 µm. D+P: diabetes+pulses.

Experimental diabetes induced a significant reduction in the axon number (particularly in large axons) and the mean axon area in the distal ON portion (Figure 3). The application of weekly ischemia pulses prevented these alterations. In the distal portion of the ON from eyes submitted to weekly ischemia pulses, the axon number and mean axon area did not differ from that observed in control animals (Figure 3).

**Figure 4:**
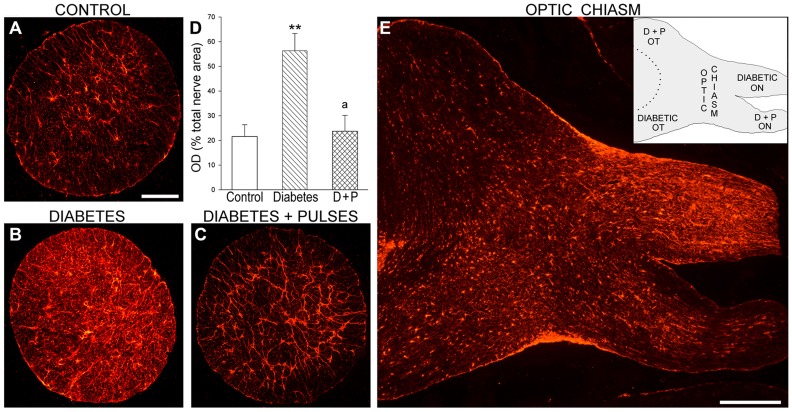
Astrocyte analysis at the distal ON portion. Representative GFAP-immunostaining in transverse sections from a control (A) and diabetic ONs from an eye without (B) or with (C) ischemic conditioning are shown. In the ON from the diabetes+sham group, the area occupied by astrocytes (measured as total optical density (OD)) was significantly increased (D). Ischemic conditioning prevented these alterations. In panel E, a horizontal section through the optic chiasm (containing part of the ON and the optic tract) from a representative diabetic rat is shown. Note a clear decrease of GFAP-immunoreactivity in ON and the optic tract from the conditioned eye as compared with the contralateral (sham-treated) pathway. The inset is a schematic representation of the horizontal section. Data are mean ± SEM (n = 6 nerves/group); ** p<0.01 versus age-matched controls, a p<0.01 versus diabetic ON from sham-treated eyes, by Tukey’s test. Scale bar: A = 200 µm, E = 600 µm. D+P: diabetes+pulses; OT: optic tract.

To investigate the effect of ischemic tolerance on diabetes-induced astrocyte alterations in the distal portion of the ON, GFAP-immunoreactivity was analyzed in diabetic rats submitted to ischemia pulses in one eye, and a sham procedure in the contralateral eye. A significant increase in GFAP-immunoreactivity in the transverse ON portion was observed in diabetic eyes submitted to a sham procedure as compared with age-matched controls, whereas ischemic tolerance decreased this parameter (Figure 4A–D). Figure 4E shows a panoramic view of GFAP-immunostaining in a horizontal section obtained at the optic chiasm level from a diabetic animal in which one eye was submitted to ischemia pulses and the other eye to a sham procedure. At the optic chiasm, most ON axons from each eye meet, cross the midline and project to the contralateral optic tract. A clear increase in GFAP-immunoreactivity was observed in the ipsilateral ON and the contralateral optic tract that corresponded to the diabetic pathway from the eye submitted to a sham procedure. In contrast, a marked decrease in GFAP-immunostaining was observed in the contralateral pathway, corresponding to the diabetic eye that received ischemia pulses.

**Figure 5:**
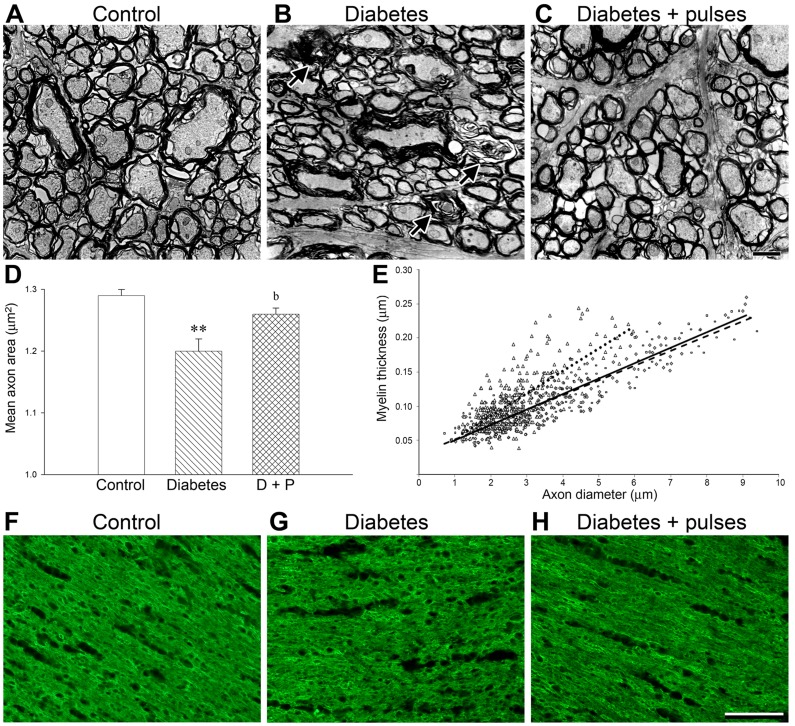
Ultrastructural analysis of myelinated axons. The distal ON portion from control (A) and diabetic ON from eyes without (B) or with ischemic conditioning (C) were evaluated. Clear signs of axon degeneration and myelin disorganization (B), and a significant decrease in the mean axon area (D) were found in the diabetic distal ON. The application of ischemia pulses (C–D) prevented these alterations. Panel E: Calculation of the axon-myelin ratio. Scatter plot displays the ratio between the myelin thickness and the respective axon diameter. The slopes were: 0.22±0.03 in control (continuous line) rats, 0.33±0.06 in diabetic (dotted line) and 0.21±0.05 in diabetes+pulses group (dashed line). Data are mean ± SEM (n = 4 nerves/group); ** p<0.01 versus age-matched controls. b: p<0.05 versus diabetic ON from sham-treated eyes, by Tukey’s test. The levels of MBP were examined in the distal portion of the ON from a control (F), a diabetic (G) and a diabetic ON from an eye submitted to ischemia pulses (H). In diabetic ON from eyes with a sham procedure, a slight disorganization of MBP-immunostaining was observed as compared with control and diabetic+ischemia pulses groups was observed. Scale bar: C = 2 µm; H = 50 µm. D+P: diabetes+pulses.

At ultrastructural level, compact bundles of myelinated axons fascicles were observed in control ONs (Figure 5A). In the diabetic distal ON, clear alterations with axon collapse and demyelination were evident (Figure 5B). The morphometric ultrastructural analysis confirmed a significant decrease in the mean axon area in the distal ON from diabetic eyes submitted to a sham procedure (Figure 5D). Ischemic conditioning significantly prevented these alterations (Figure 5C–D). Scatter plots display the ratio myelin thickness/axon diameter (Figure 5E). In the diabetic ON submitted to a sham procedure, the reduction in axon diameter was more pronounced in large axons, which resulted in a significant increase in the plot slope, as compared with control eyes (*P*<0.01) and diabetic eyes subjected to ischemia pulses (*P*<0.01). The plot slope in diabetic ONs from eyes submitted to ischemia pulses did not differ from that obtained in control (non-diabetic) ONs. Figure 5 also shows an analysis of MBP (a reliable index of OL differentiation and myelin formation) in horizontal sections of the distal ON portion. A slight disorganization of MBP-immunostaining was observed in sham-treated ONs from diabetic animals (Figure 5G) as compared with non-diabetic animals (Figure 5F), whereas ischemic pulses prevented these changes (Figure 5H).

**Figure 6:**
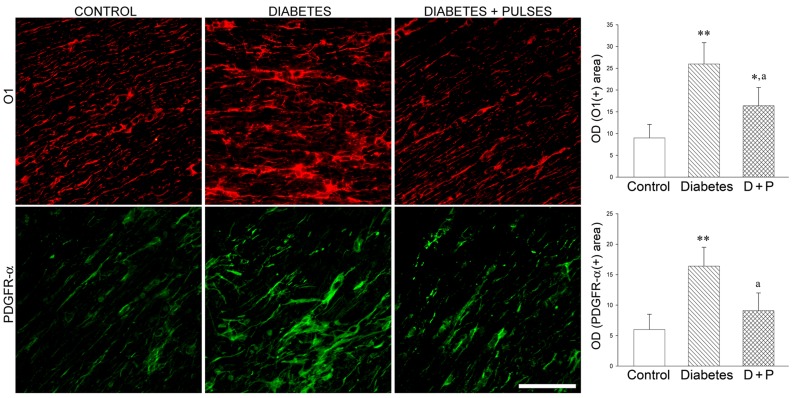
OL linage evaluation. Immature OL (O1+ cells) and OL precursor (PDGFR-α+ cells) were evaluated by immunostaining in transverse ON sections. In the diabetic ON from eyes that received a sham treatment, a significant increase in O1(+) and PDGFR-α (+) area was observed, with the presence of disorganized and hypertrophic cells. In the right panel, the area occupied by glial cells (measured as total optical density (OD)) is shown. Ischemic conditioning significantly prevented these alterations and a clear decrease in O1- and PDGFR-α-immunoreactivity, with cells aligned parallel to axon bundles were found. Data are mean ± SEM (n = 6 nerves/group); * p<0.05, ** p<0.01 versus age-matched controls, a p<0.01 versus diabetic ON from sham-treated eyes, by Tukey’s test. Scale bar = 50 µm. D+P: diabetes+pulses.

In order to evaluate the effect of ischemic tolerance on OL-linage, the effect of ischemia pulses on the population of immature OL (O1+) and OL precursor (PDGFRα) cells in diabetic eyes was analyzed. In the diabetic distal ON portion, an increase in O1- and PDGFRα-immunoreactivity, with hypertrophic somas and a high number of processes were found (Figure 6). The application of ischemia pulses prevented these alterations. A clear decrease in O1- and PDGFRα-immunoreactivity in cells aligned parallel to axon bundles was observed in diabetic eyes submitted to ischemia pulses (Figure 6).

**Figure 7:**
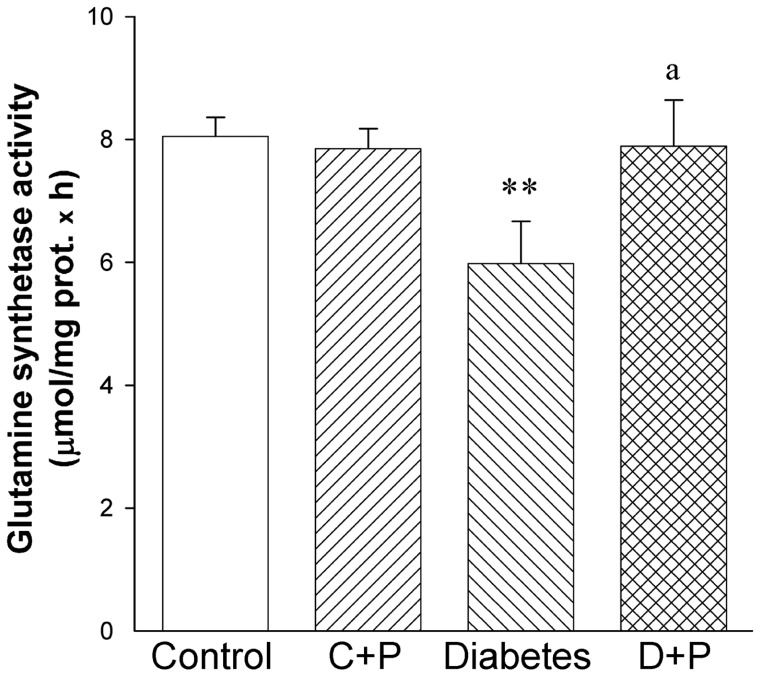
Effect of diabetes and ischemic conditioning on glutamine synthetase activity. In sham treated retinas from diabetic animals, a significant decrease in GS activity was observed, whereas the application of ischemic conditioning prevented this alteration. Data are mean ± SEM (n = 8 retinas/group). ** p<0.01 versus age-matched controls, a p<0.01 versus diabetic sham-treated eyes, by Tukey’s test. C+P: control+pulses; D+P: diabetes+pulses.

In order to analyze the mechanism involved in the protection induced by ischemic conditioning on the diabetic optic pathway, retinal GS activity was assessed at 6 weeks of diabetes onset. In diabetic eyes submitted to a sham procedure, a significant decrease in GS activity as compared with control retinas was observed, whereas ischemia pulses significantly prevented the decrease in this parameter (Figure 7).

## Discussion

The present results indicate that ischemic conditioning, which showed no effect *per se*, prevented a deficit in the anterograde transport from the retina to the SC, and the decrease in axon number and myelination, as well as the alterations in astrocytes and OL lineage in the distal portion of the ON induced by experimental diabetes.

Evidence of axon degeneration occurring separate from, and before soma degeneration has been described in many neurodegenerative diseases, such as Alzheimer disease, Parkinson disease, and glaucoma [23]–[26]. In agreement, we have shown significant axoglial alterations in the distal portion of the ON at an early stage of experimental diabetes, which precede RGC loss [7].

Several lines of evidence strongly support that ischemic tolerance induced by IPC or PostC is an important phenomenon of the central nervous system adaptation to subsequent or previous ischemia, respectively. In fact, ischemic conditioning is actually considered the strongest known procedure to prevent or avoid neurodegeneration. Ischemic tolerance works specifically in vulnerable neuronal populations, such as pyramidal neurons in the hippocampal CA1 region [27] and in other brain cell populations [28], including retinal cells [19], [20]. However, the beneficial effect of ischemic tolerance against central nervous system axon alterations was not previously examined. Since at an early stage of STZ-induced diabetes (i.e. 6 weeks after STZ injection) axon degeneration occurs while RGC somas survive, this could be a suitable model to analyze the effect of ischemic tolerance on axonal damage.

As shown herein, ischemic conditioning prevented the deficit in the CTB anterograde transport from the retina to the SC, its primary projection site, indicating that ischemic pulses prevented a “misconnection” between the retina and the SC induced by experimental diabetes. In animals that had been diabetic for 6 weeks, although no obvious changes in retinal morphology and in the number of RGCs were observed, a large increase in astrocyte reactivity (as shown by GFAP-immunoreactivity) occurred in the distal portion of the ON, which coincided with a significant axon loss, being large axons significantly more vulnerable than small axons. The application of ischemia pulses prevented the increase in GFAP-immunoreactivity, and the decrease in axon number and mean axonal area.

It is well established that the first site of neuronal damage may be remote from the site of insult 29]. For example, in transected motor axons, neuromuscular junctions that are many centimeters from the lesion degenerate first, while the axons immediately adjacent to the lesion remain intact for two to three times longer than the distant terminals [30]. Thus, the fact that axoglial alterations at the distal portion of the ON seems to be the first structural alteration in the diabetic visual pathway does not implies that the initial damaging event occurs at axonal level. Instead, a putative sublethal impairment of RGC soma could render them unable to support their axons, and concomitantly induce glial (astrocytes and OL) alterations, resulting in progressive dying-back of the axon toward the cell body [29]. At present, we could not ascertain whether the damage induced by experimental diabetes, and the protection provoked by ischemic conditioning primarily occurred in RGC soma or in the ON. However, considering that ischemia pulses affect primarily the retina, it seems likely that diabetic damage of the ON begins at retinal levels. In this scenario, changes in retinal neurochemistry could be involved both in the ON damage induced by experimental diabetes, as well as in the protection induced by ischemic conditioning.

Glutamate is the main excitatory neurotransmitter in the mammalian retina, but it is neurotoxic when present in excessive amounts. Müller cells surround glutamatergic synapses, and express glutamate transporters, and the glutamate metabolizing enzyme, GS. Extracellular glutamate is transported into Müller cells and amidated by GS to the non-toxic amino acid glutamine, which is then released by Müller cells and taken up by neurons, where it is hydrolyzed by glutaminase to form glutamate again. Thus, Müller cells play a substantial role in the retinal glutamate/glutamine cycle activity, and their role in maintaining low extracellular concentrations of glutamate may be particularly critical in diabetes [31]. In that sense, elevated levels of glutamate in the retina have been found in experimental models of diabetes as well as in the vitreous fluid of diabetic patients [32]–[34]. The local concentration of glutamate at the membrane receptors, but not high levels of glutamate in the vitreous, is a necessary condition for excitotoxicity to be involved in diabetic neuropathy. However, there is sparse information about this issue in early stages of DR [35]. High levels of glutamate in DR have been related to a dysfunction in metabolizing glutamate [33]. In fact, several lines of evidence support that retinal GS could be a target for diabetic damage. Significant differences in GS at early stage of diabetes, as shown by real-time PCR, Northern blot analysis, and enzyme activity were confirmed by several groups [33], [36], [37]. In agreement, we found a significant decrease in GS activity at 6 weeks of diabetes onset, which was prevented by ischemia pulses. The effect of ischemic conditioning on the decrease in GS activity, could suggest that it could behave as an anti-excitotoxicity therapy, avoiding excessive accumulation of glutamate, protecting neurons, and consequently, inducing axonal recovery.

The past two decades have provided interesting insights into the mechanisms and potential applications of ischemic tolerance which can protect neurons and improve neuronal survival after ischemia or even against several types of non-ischemic damage. These results suggest that early vision loss in diabetes could be abated by ischemic conditioning which preserved distal axonal function and structure before the neuronal soma loss. Moreover, the present results add new potentialities to the therapeutic effects of ischemic tolerance, which is axon protection. Thus, ischemic tolerance could have promise for application in other neurodegenerative axonal diseases.

## Materials and Methods

### Ethics Statement

All animal procedures were in strict accordance with the ARVO Statement for the Use of Animals in Ophthalmic and Vision Research. The ethic committee of the School of Medicine, University of Buenos Aires (Institutional Committee for the Care and Use of Laboratory Animals, (CICUAL)) approved this study.

### Animals

Male *Wistar* rats (average weight, 300–350 g) were housed in a standard animal room with food and water *ad libitum* under controlled conditions of humidity and temperature (mean ± SD, 21±2°C), and under a 12-hour light/12-hour dark lighting schedule (lights on at 8∶00 AM).

For diabetes induction, a single dose of STZ (Sigma-Aldrich, St. Louis, MO), 60 mg/kg (in 0.1 mol/L citrate buffer, pH 4.5), was i.p. injected. Control rats received an equal volume of citrate buffer. The animals were examined 72 hours after injection using a glucose meter (Contour TS, Bayer, Buenos Aires, Argentina), and those with blood glucose levels greater than 350 mg/dL were considered diabetic. Body weight and plasma glucose level were determined weekly. For the assessment of body weight and blood glucose levels, a group of diabetic animals with intact eyes was included.

### Ischemic Conditioning Protocol

Retinal ischemia was induced as previously described [21]. In brief, animals were anesthetized with ketamine hydrochloride (150 mg/kg) and xylazine hydrochloride (2 mg/kg), and after topical instillation of proparacaine, the anterior chamber was cannulated with a 30-gauge needle connected to a pressurized bottle, filled with sterile saline solution. Retinal ischemia was induced by increasing intraocular pressure (IOP) to 120 mm Hg for exactly 5 min. The contralateral eye was cannulated without raising IOP (sham procedure). This maneuver was first applied after confirming the high blood glucose levels and then was weekly repeated. In a group of non-diabetic animals, only one eye was weekly subjected to 5-min ischemia, whereas the contralateral eye remained intact.

### Cholera Toxin β-subunit Transport Study

Rats were anesthetized nd a drop of proparacaine (0.5%) was topically administered for local anesthesia. Four microliters of a solution of 0.2% cholera toxin β-subunit (CTB) conjugated to Alexa 488 dye (Molecular Probes, OR, USA) in 0.1 M PBS (pH 7.4) was injected into the vitreous using a 30-gauge Hamilton syringe (Hamilton, Reno, NV, USA). Injections were applied at 1 mm of the limbus and the needle was left into the eye for 1 minute to prevent volume loss. At 3 days after injection, rats were anesthetized and intracardially perfused with saline solution followed by a solution containing 4% formaldehyde in 0.1 M PBS (pH 7.4). Their brains were carefully removed, post-fixed overnight at 4°C, and immersed in a graded series of sucrose solutions (10%, 20%, and 30%); coronal sections (40 µm) were obtained using a freezing microtome (Leica Microsystems Inc., Buffalo Grove, IL). Nuclei were stained with fluorescent dye 4′,6-diamidino-2-phenylindole (DAPI), mounted with antifade medium (Vectashield; Vector Laboratories, CA, USA) and viewed with a fluorescence microscope (BX50; Olympus, Tokyo, Japan) connected to a video camera (3CCD; Sony, Tokyo, Japan) attached to a computer running image analysis software (ImagePro Plus, Media Cybernetics). For immunofluorescence studies, comparative digital images from different samples were grabbed using identical exposition time. Digitalized captured TIF-images were finally assembled and processed in Adobe Photoshop SC (Adobe Systems, San Jose, CA). For illustration purposes, the image quality was optimized by making adjustments in brightness and contrast that were constantly applied to all images. No other adjustment was made. All the nomenclature used in the paper follows that of Paxinos and Franklin [38].

CTB staining was quantified as previously described [25] with some modifications. Coronal sections (every other cut, approximately 30–35 sections) were used for the SC reconstruction using Matlab (Math Work). For each section, the retino-recipient SC was outlined using DAPI-counterstaining and the total retinotopic area was calculated. Digital images were converted to 8-bits of grey scale and the optic density of CTB-staining was calculated. The total length was measured and divided in bins (4 µm) from the medial to lateral region. The CTB density was obtained by dividing the total pixel area by CTB^+^ pixels. Finally, a colorimetric thermal representation was applied (from 0% = blue to 100% = red). The number of sections and the thickness (2x) were used for a final reconstruction of the retinal projection to the SC. Five animals per group were used.

### Histological Examination

Anesthetized rats were intracardiacally perfused as previously described herein. Then, eyes and brains were carefully removed and immersed for 24 h in the same fixative. Eyes were embedded in paraffin and sectioned (5 µm) along the vertical meridian through the ON head. Sections were stained with hematoxylin and eosin and used for morphometric analysis. Digital images were obtained at 1 mm dorsal and ventral from the optic disk. The total retinal, inner plexiform layer, inner nuclear layer and outer nuclear layer thickness (in µm) was measured for each eye. For each eye, results obtained from four separate sections were averaged and the mean of 10 eyes was recorded as the representative value for each group. For Brn3a immunostaining, retinas were isolated and flat-mounted with the vitreous side up in glass slides.

ON portions at 2 mm before the optic chiasm (intracranial portion) were dissected out and after several washings, tissue blocks were postfixed in 2% osmium tetroxide in sodium phosphate buffer for 1 hour. Dehydration was accomplished by gradual ethanol series, and tissue samples were embedded in epoxy resin. Semithin sections (1 µm) were obtained with an ultramicrotome, stained with toluidine blue and used for morphometric analysis. In some cases, the ONs were dehydrated and embedded in paraffin. Transverse and longitudinal sections (7 µm) were obtained and used for immunostaining analysis. ON total area was calculated from light microphotographs of transverse semithin sections obtained with a final magnification of 20x. The nerve was divided in four quadrants and the number and area of axons were calculated in each section (in areas randomly chosen in each quadrant) from micrographs obtained at 100x. Images were converted to 8-bits grey scale and a manual threshold value, first determined by visual examination, was constantly applied. Finally, images were converted to a binary form. Myelinated fiber sizes were digitally delineated by the outer margin of their myelin sheaths and axon areas were measured. For each ON, results obtained from four separate sections were averaged, and the mean axon number and area of six nerves was recorded as the representative value for each group.

The brains were coronally sectioned (behind the optic chiasm) and the anterior portion was included in paraffin as previously described [39]. Horizontal sections (10 µm) were obtained at the level of the optic chiasm (containing the ON and the optic tract portions). The posterior portion of the brain was immersed in a graded series of sucrose solutions (10%, 20%, and 30%) and coronal sections (40 µm) were obtained using a freezing microtome.

### Immunoflourescence Protocol

Sections were immersed in 0.1% Triton X-100 in 0.1 M PBS for 10 min. Antigen retrieval was performed in paraffin sections, by heating (90°C) slices for 30 minutes in citrate buffer (pH 6.3). Sections were pre-incubated with 5% normal horse serum for 1 h, and then incubated overnight at 4°C with the following primary antibodies: a mouse monoclonal anti-glial fibrillary acidic protein (GFAP) antibody conjugated to Cy3 (1∶800; Sigma Chemical Co., MO, USA), a rabbit polyclonal anti-myelin basic protein (MBP) antibody, a mouse polyclonal IgM O1 antibody (the anti-MBP and O1 antibody were generously given by Dr. Campagnoni (Mental Retardation Research Center, University of California, Los Angeles, CA, USA)), a goat polyclonal anti-platelet-derived growth factor receptor-α (PDGFR-α) antibody (1∶100; Neuromics, MN, USA and a goat polyclonal anti-Brn3a antibody (1∶500; Millipore, USA). After several washings, a donkey anti-mouse IgM secondary antibody conjugated to Cy3 (1∶200; Jackson ImmunoResearch. PA, USA), and a donkey anti-Goat secondary antibody conjugated to DyLight 488 (1∶100; Jackson ImmunoResearch, PA, USA) and a goat anti-rabbit secondary antibody conjugated to Cy2 (1∶100; Jackson ImmunoResearch, PA, USA) were added, and sections were incubated for 2 h at room temperature. Regularly, some sections were treated without primary antibodies to confirm specificity. After immunostaining, nuclei were stained with DAPI, mounted with antifade medium and viewed with a fluorescence microscope as described.

For all morphometric image processing and analysis, digitalized captured TIF-images were transferred to ImageJ software (NIH, USA). For immunostaining density analysis and area determination (percentage of area occupied by glial processes) in ON sections, images were converted to 8-bits grey scale and in each case, a manual threshold value was determined to binarize the images. Results obtained from three sections were averaged and recorded as the representative value for each ON. Six animals per group were used. For RGCs density determination, the retina was divided in 4 quadrants, and images from the central and peripheral area were obtained. The number of Brn3a(+) cells was measured and expressed as number of cells/mm^2^. For each group, results obtained from four separate quadrants were averaged, and the mean of 5 eyes was recorded as the representative value.

### Electron Microscopy

After anesthesia and thoracotomy, animals were perfused through the left ventricle with a fixative solution containing 2% glutaraldehyde and 4% formaldehyde in 0.1 M PBS (pH 7.4). Semithin sections were obtained as described above and ultrathin sections were stained with uranyl acetate and lead citrate. Finally, sections were viewed and photographed using a Zeiss EM 10 C transmission electron microscope. Electron microscopical photographs were obtained with a final magnification of 3000 times. Axon areas were calculated from approximately 10 photographs (25×25 µm) per nerve. The calculation of axon-myelin ratio was obtained from no fewer than 200 myelinated axons per nerve. Data from four nerves were averaged and recorded as the representative slope value for each group.

### Glutamine Synthetase Activity Assessment

Each retina was homogenized in 200 µl of 10 mM potassium phosphate, pH 7.2. Glutamine synthetase (GS) activity was assessed as described [40]. Reaction mixtures contained 150 µL of retinal homogenates and 150 µl of a stock solution (100 mM imidazole-HCl buffer, 40 mM MgCl_2_, 50 mM β-mercaptoethanol, 20 mM ATP, 100 mM glutamate, and 200 mM hydroxylamine, adjusted to pH 7.2). Tubes were incubated for 15 min at 37°C. The reaction was stopped by adding 0.6 ml of ferric chloride reagent (0.37 M FeCl_3_, 0.67 M HCl, and 0.20 M trichloroacetic acid). Samples were placed for 5 min on ice. Precipitated proteins were removed by centrifugation, and the absorbance of the supernatants was read at 535 nm against a reagent blank. Under these conditions, 1 µmol of γ-glutamylhydroxamic acids gave an absorbance of 0.340. GS specific activity was expressed as µmoles of γ-glutamylhydroxamate per hour per milligram of protein. Data from eight retinas were averaged and recorded as the representative value for each group.

### Statistical Analysis

Statistical analysis of results was made by a two-way analysis of variance (ANOVA) followed by Tukeýs test.
